# Viral Mastery: The Dynamic Regulation of Interferon Signaling

**DOI:** 10.3390/v18060674

**Published:** 2026-06-16

**Authors:** Niranjan Dodantenna

**Affiliations:** College of Veterinary Medicine, Chungnam National University, Daejeon 34134, Republic of Korea; niranjan3k@gmail.com

**Keywords:** innate immunity, interferons, JAK-STAT, virus proteins, immune evasion

## Abstract

Interferons (IFNs) are antiviral cytokines that serve as key mediators of the innate immune response, and their production is induced in the majority of cells within hours of pathogen entry. IFNs are predominantly produced by pathogen-infected cells; however, their antiviral effects extend to surrounding cells through autocrine and paracrine signaling mechanisms, inducing the transcription of hundreds of antiviral genes. Numerous gene products either interfere directly with viral replication or play regulatory roles that influence the progression and strength of the ensuing immune response. Viruses, on the other hand, have devised techniques to circumvent the host antiviral immune response and establish infection. This review focuses on the current state of evidence demonstrating how certain viral proteins block antiviral responses via immunomodulatory strategies and discusses how to overcome these immune evasion tactics.

## 1. Introduction

Innate immune responses are the first line of defense against viral infections. Pattern recognition receptors (PRRs) on host cells recognize conserved pathogen features, which recruit several adaptor proteins to signal downstream and trigger the IFN response [1]. The IFN system is present in all vertebrates and plays a critical role in antiviral responses [2]. Isaacs and Lindenmann identified a broad family of IFN proteins in 1957 and named them for their ability to “interfere” with viral replication [3]. In general, viral infections cause the transient production and secretion of IFNs to protect uninfected cells. IFNs trigger the transcription of hundreds of IFN-stimulated genes (ISGs), which encode proteins that impede many viral replication processes [4]. Specific ISG proteins are particularly efficient against specific virus families, and maximum protection is achieved by inhibiting more than one step of virus replication using distinct ISG proteins.

Mammalian IFNs are classified into three groups—type I, type II, and type III—according to their receptor usage, induction mechanisms, biological functions, and amino acid sequence characteristics [5]. Each IFN class—type I, II, and III—prompts signals via a unique heterodimeric receptor and stimulates gene expression via the Janus kinase-signal transducer and activator of transcription (JAK-STAT) signaling pathway. Type I IFNs were first identified based on their antiviral activity [6]. However, its role in bacterial infections is more complex and exhibits multifaceted biological activities [7,8]. Several IFN-α subtypes (13 in humans and 14 in mice), as well as a single IFN-β, IFN-ε, IFN-κ, IFN-ω (humans), and IFN-ξ (mice) subtypes, are included in type I IFNs [6]. IFN-γ is the sole member of the type II IFN class in mammals, whereas teleost fish possess two type II IFN members [8,9]. In 2003, two groups discovered type III IFNs (IFN-λ1, IFN-λ2, and IFN-λ3) that were structurally similar to interleukin-10 (IL-10) [10,11]. IFN-λ4, a type III IFN, was discovered in 2013 and linked to the reduced clearance of hepatitis C virus (HCV) infection [12]. Recently, Chen et al. reported the discovery of a novel type IV IFN (IFN-υ) system in jawed vertebrates. This system is associated with the induction of IFN-υ-stimulated genes and antiviral/antibacterial responses, expanding our understanding of the evolution and functional diversity of vertebrate IFN-mediated immunity [13,14,15].

It is not surprising that viruses adopt various tactics to increase their replication. Numerous viruses, including the influenza virus, West Nile virus (WNV), yellow fever virus (YFV), vesicular stomatitis virus (VSV), dengue virus (DENV), herpes simplex virus (HSV), and several HCVs, are inhibited by type I IFN signaling in humans [16,17]. Thus, evading type I IFN signaling is critical for viruses to successfully replicate within their hosts, as demonstrated by many flaviviruses; Zika virus (ZIKV), Japanese encephalitis virus (JEV), WNV, and DENV can all antagonize type I IFN signaling in human cells to promote their genome replication [18].

Several reviews have summarized viral strategies that antagonize IFN-induced responses, focusing on the general mechanisms of viral immune evasion [19,20,21,22], virus family-specific IFN antagonism [23,24,25], or viral targeting of selected components of the IFN signaling pathway [26,27]. These studies have significantly advanced our understanding of virus–host interactions by describing how viruses interfere with IFN induction, signaling, and antiviral effector functions. While previous reviews have largely focused on specific aspects of IFN antagonism, this review provides a comprehensive analysis of how diverse virus families and viral proteins employ multiple strategies to target IFN signaling components, exerting conserved and virus-specific strategies. This review summarizes the existing knowledge of how diverse viral proteins evade IFN-induced immune responses by targeting key components of the IFN signaling pathway, from IFN receptors (IFNRs) to IFN-stimulated response element (ISRE) promoters, establishing a permissive environment for viral replication within the host. Furthermore, this review emphasizes the functional redundancy and cooperative action of multiple viral proteins within the same virus in disrupting both classical and non-classical IFN signaling pathways, highlighting the complexity of immune evasion by viruses. It underscores the amino acid-level interactions between viral proteins and host IFN signaling molecules, integrating information dispersed across the literature into a single, comprehensive resource that will support virologists and immunologists in guiding future research directions.

## 2. The IFN-Induced Signal Transduction Mechanism and Receptor-Dependent Regulation

The primary function of IFNs is to alert uninfected cells to the presence of an incoming virus, enabling them to initiate a defensive response before infection. Following synthesis and secretion, IFNs exert their biological effects by binding to their cognate cell surface receptor complex, causing conformational alterations in the intracellular domain, and triggering a series of post-translational modifications (PTMs) of intracellular signaling molecules. There are only three IFNRs on the cell surface, despite the presence of approximately 20 IFNs.

The type I IFN receptor (IFNAR) consists of two subunits: IFN α receptor 1 (IFNAR1) and IFNAR2. The type II IFN receptor (IFNGR) is composed of IFN γ receptor 1 (IFNGR1) and IFNGR2. The type III IFN receptor (IFNLR) is formed by the IFN λ receptor 1 (IFNLR1) and interleukin-10 receptor β subunit (IL-10R2). Although each IFN type signals through a distinct receptor complex, types I and III activate overlapping downstream signaling pathways and induce similar ISGs [28,29]. Conversely, studies by Forero et al. and the extensive review by Lazear et al. demonstrated that type I and III IFNs perform distinct functions. Differences in IFNR localization and expression enable type III IFNs to mainly restrict viral replication at mucosal and infected tissues while minimizing tissue damage, whereas type I IFNs induce IRF1-mediated chemokine expression that recruits immune cells and promotes antiviral protection. Therefore, type I IFN (IFN-β) induces a rapid but short-lived ISG response, whereas type III IFN (IFN-λ3) elicits a delayed but prolonged ISG induction. Through these distinct patterns of ISG regulation, type I and type III IFNs exert non-redundant antiviral functions that contribute to sustained resistance to viral infection [30,31]. Only IFNGR has one ligand among the three receptors: IFN-γ [32].

Once the ligand binds to the dimeric receptor complex of IFNAR, the receptor-related kinases, tyrosine kinase 2 (TYK2) and Janus kinase 1 (JAK1), are auto-phosphorylated and then phosphorylate tyrosines in the cytoplasm of the receptor. The IFNAR subunits are then phosphorylated on tyrosine, creating a docking site for the STAT1 and STAT2 transcription factors to bind. This causes the recruitment and phosphorylation of STAT1 and STAT2 proteins. Following activation, STAT1 and STAT2 interact with interferon regulatory factor 9 (IRF9) to create ISG factor 3 (ISGF3). Next, the ISGF3 complex translocates into the nucleus and binds to ISG promoters containing an ISRE, causing the associated genes to be induced [33,34]. IFNLR uses the same JAK-STAT signaling pathway to create a similar pool of ISGs [35]. In contrast, IFNGR is unable to recruit TYK2 and does not activate STAT2. As a result, the STAT1 homodimer or gamma interferon activation factor (GAF), the major transcription factor activated by type II IFNs, activates the transcription of genes with gamma-activated sites (GAS) in their promoters [36]. When ISRE and GAS are activated, transcription of ISGs, such as OAS, GBPs, NOS2, IFITMs, TRIMs, Viperin, ISG15, MXs, IRFs, STATs, IFNs, and others is initiated [17]. To prevent viral multiplication in host cells, these ISGs target the viral structure, attachment, entrance, viral gene expression, viral protein translation, viral genome replication, viral assembly, and viral release [37].

## 3. Viral Strategies to Evade IFN Signaling

**Figure 1:**
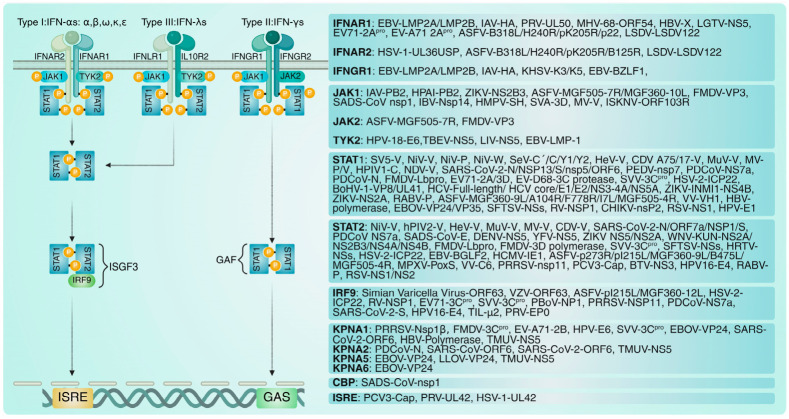
Schematic overview of viral factors that evade key components of the IFN signaling pathway.

Hosts have evolved sophisticated mechanisms to maintain balanced IFN responses, whereas viruses have developed diverse strategies to counteract these host defenses. As illustrated in Figure 1, viral proteins target multiple stages of IFN signaling, including gene transcription, mRNA translation, PTMs, protein localization, and signaling complex assembly. By disrupting these regulatory processes, viruses suppress antiviral responses, enhance immune evasion, and contribute to their virulence.

**Figure 2:**
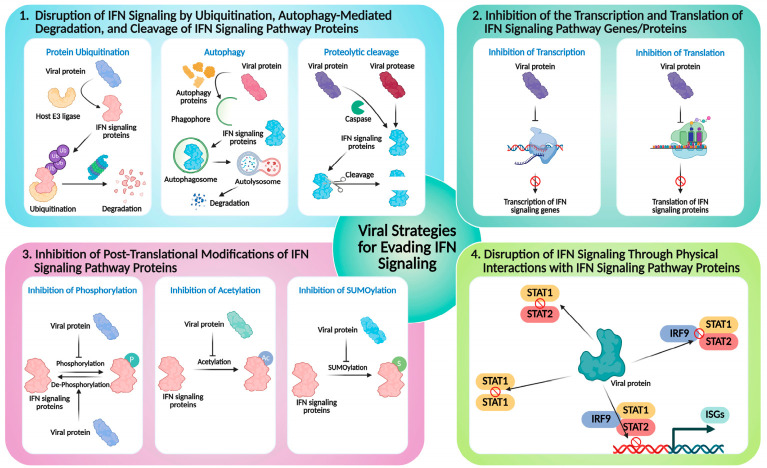
Mechanisms of Viral Evasion of IFN Signaling.

Viruses counteract the effects of IFNs using ubiquitin-proteasome and autophagy mechanisms, leading to the degradation of essential signaling molecules in the IFN pathway [38,39]. Moreover, viral proteases can cleave key proteins involved in the IFN pathway, either directly or through caspase-dependent mechanisms (Figure 2, top left panel) [40,41].

Eukaryotic cells synthesize mRNA in the nucleus and translate it in the cytoplasm. Viruses evade the host defense mechanisms by interfering with the processes of mRNA synthesis, processing, and stability in several ways, including interference with the activity of RNA polymerase enzymes in cells, mRNA capping, splicing, termination of transcription, polyadenylation, and cleavage [42]. Cap-dependent mRNA translation requires the eIF4F complex (eIF4E, eIF4A, eIF4G), where eIF4G-PABP interaction links the 5′ and 3′ ends. Many viruses disrupt these steps to block translation, but few viral proteins are known to inhibit transcription and translation of IFN signaling genes (Figure 2, top right panel) [42,43,44,45,46,47].

PTMs modulate immune responses by influencing protein folding, stability, subcellular localization, signaling, and interactions with other molecules [48,49,50]. The antiviral response relies on PTMs, such as phosphorylation, ubiquitination, and others like SUMOylation, methylation, and acetylation [48,51]. As depicted in Figure 2, bottom left panel, viruses make use of PTMs by degrading important proteins in IFN signaling via ubiquitination or by modifying the phosphorylation state of such proteins, thereby disrupting IFN signaling [52,53,54].

Viruses can inhibit the IFN response by interfering with the signaling components directly to inhibit either the formation of the dimerizing complex, ISGF3, or the translocation into the nucleus. Thereby, viruses block the formation of functional signaling complexes and ISGs (Figure 2, bottom right panel) [55,56,57].

## 4. Downregulation of IFN Receptor Signaling

The type I IFN ligand binds with its IFNAR1, causing receptor dimerization (IFNAR1-IFNAR2). The interaction between the ligand and the receptor causes JAK to be trans-phosphorylated. JAK activation leads to the tyrosine phosphorylation of the bound receptor, creating a docking site for STATs. JAK phosphorylates STAT at this docking location, and STAT dissociates from the receptor to create homodimers or heterodimers via SH2-domain-phosphotyrosine interactions [58]. The amount of IFNAR1 protein has been demonstrated to be critical for triggering the downstream signaling pathway mediated by JAK/STAT [59].

From the virus’s perspective, it is critical to escape the host’s innate immune response during the early stages of infection. Since IFNR initiated the JAK-STAT signaling pathway in response to virus-induced IFNs produced, many viruses possess proteins that interfere with IFNR degradation. For example, Epstein–Barr virus (EBV) latent membrane protein 2A (LMP2A) and 2B (LMP2B) degrade intracellular IFNAR1 and IFNGR1 without interfering with their cell surface expression, though the mechanism of the degradation is yet to be resolved. The findings suggest that internalized IFNRs may be directed into a degradative arm of the endocytic pathway by partial co-localization of LMP2A and LMP2B with IFNRs in late endosomes and lysosomes [60]. Influenza A virus (IAV) infection triggers phosphorylation and the K48- and K63-linked ubiquitination of IFNAR1. Mechanistically, the HA protein of IAV was shown to reduce the surface level of IFNAR1. Notably, the HA1 subunit, but not HA2, promotes IFNAR1 ubiquitination, leading to its degradation through both proteasomal and lysosomal pathways [61]. Another study showed that IAV-HA prompts IFNAR1 degradation mediated by Casein kinase 1 α (CK1α) [62], which was previously demonstrated to be required for phosphorylation and downregulation of IFNAR1 in response to ER stress and viral infection [63]. Moreover, Pseudorabies Virus (PRV) 225–253 residues in the C-terminal region of UL50 protein accelerate lysosomal degradation of IFNAR1 independent of its dUTPase activity [64]. ORF54 of the murine gammaherpesvirus-68 (MHV-68) is another dUTPase that degrades IFNAR1 independently of its enzymatic activity [65]. The African swine fever virus (ASFV) p22 protein promotes the interaction of IFNAR1 with Tax1-binding protein 1 (TAX1BP1) through its transmembrane domain (TMD), leading to autophagic degradation of IFNAR1 and suppression of the JAK-STAT signaling pathway. The p22 TMD mediates binding to both IFNAR1 and TAX1BP1, while IFNAR1 interacts with p22 via its TMD, and the CC region of TAX1BP1 is essential for its association with p22 [38]. Another protein of ASFV, pB125R and its N and C-terminal, binds to the TMD of IFNAR2 (aa 247–266), promoting its autophagic degradation, impairing the signal transduction of the IFN response at an early stage. This ultimately reduces the nuclear translocation of the ISGF3 complex and decreases ISG production [66].

Some viral proteins have been shown to interfere with the transcription and translation of IFNR components, thereby suppressing the induction of ISGs. For instance, A previous study suggested that hepatitis B virus (HBV) X protein (HBX), a regulatory protein of HBV, activates STAT1, leading to type I IFN production in Chang liver carcinoma cells. Type I IFN released by HBX-expressing hepatocytes strengthens antiviral signals by interacting with the homologous type I IFNR [67]. HBX protein handles this detrimental situation by downregulating IFNAR1 by decreasing its expression and increasing IFNAR1 translocation into the cytoplasm, although a proper mechanism of action remains elusive [68]. The interaction of the Langat virus (LGTV) NS5 protein with prolidase (PEDP) 184–255 residues inhibits IFNAR1 surface expression. R376A, E626A, V1630/1631A, and W647A mutations of NS5 result in disruption of interaction with IFNAR1. Neither ER stress nor cholesterol redistribution is necessary for this downregulation [69]. Enterovirus 71 (EV71) 2A^pro^ has been demonstrated to diminish the IFNAR1 expression. 2A^pro^-C110A mutation shows that it deploys its protease activity for the downregulation [70]. Mechanistically, EV-A71 2A^pro^ reduces IFNAR1 translation by cleaving the eIF4GI, without changing IFNAR1 mRNA levels generated by IFN-α. This cleavage could be mediated by the activation of caspase-3 by 2A^pro^, as the inhibition of caspase-3 activation resulted in the partial restoration of IFNAR1 in cells transfected with 2A^pro^ or infected with EV-A71 [46].

Several ASFV proteins have been shown to interact with IFNARs to interfere with their adapter proteins. For example, ASFV B318L, a transgeranylgeranyl-diphosphate synthase (GGPPS) and H240R proteins, associated with IFNAR1 and IFNAR2 to block IFNAR1-TYK2 and IFNAR2-JAK1 interaction, suppressing downstream IFN signaling [71,72]. The suppression of IFN-α-induced ISG expression by pB318L appears to depend on its GGPPS enzymatic activity, with D129 contributing to the proper functioning of this enzyme. Another study revealed that the ASFV protein pK205R interacts mostly with the intracellular domain of IFNAR1 and IFNAR2 (which is associated with TYK2 and JAK1) through specific amino acid residues Q18, N25, K32, N43, R47, T143, and S145. This interaction disrupts the association of IFNAR1/2 with the kinases JAK1 and TYK2, thereby blocking the phosphorylation and nuclear translocation of STAT proteins [73]. HSV-1 ubiquitin-specific protease UL36USP protein has been shown to interact with IFNAR2, competing with JAK1, ultimately abrogating ISG transcription [74]. Lumpy skin disease virus (LSDV) is a pathogenic virus of significant concern. Recent studies have identified the LSDV122 protein as an antagonist of type I IFN-mediated innate immune responses. Mechanistically, LSDV122 interacts with IFNAR1 and IFNAR2, thereby disrupting proper receptor assembly and preventing the recruitment of the downstream kinases JAK1 and TYK2. This interference impairs IFN-β-induced JAK-STAT signaling and consequently suppresses the expression of antiviral ISGs. Further analyses revealed that LSDV122 specifically associates with IFNAR1 and IFNAR2 deletion mutants containing both TMD and cytoplasmic domain, while the TMD of LSDV122 itself is essential for mediating these interactions [75].

In response to virus-induced IFN-γ, IFNGR activates the JAK-STAT signaling pathway, and a few viral proteins have been shown to interfere with IFNGR-mediated ISG production. Similar to the degradation of IFNAR1, LMP2A, and LMP2B of EBV degrade intracellular IFNGR1 without interfering with its cell surface expression [60], albeit the mechanism of degradation is unknown. IAV-HA causes IFNGR phosphorylation, ubiquitination, and lysosome-dependent degradation, which is mediated by CK1α [62]. Also, K3 and K5 proteins of Kaposi’s sarcoma-associated herpesvirus (KSHV) preferentially target IFNGR1 and induce its ubiquitination, endocytosis, and degradation, resulting in decreased IFNGR1 surface expression. K5 appeared to suppress IFNGR1 more strongly than K3, and the amino-terminal ring finger motif and carboxyl-terminal area of K5 are required for IFNGR1 suppression [52]. EBV immediate-early protein BZLF1 inhibits the transcription and translation of IFNGR, blocking IFN-γ-induced STAT1 tyrosine phosphorylation and nuclear translocation [43].

The studies considered here also highlight several emerging notions and unresolved issues. First, unrelated viruses repeatedly hijack common host pathways, such as ubiquitin-mediated degradation, lysosomal trafficking, and autophagy. These cellular processes represent conserved vulnerabilities that could be therapeutically targeted. Second, although many viral proteins have evolved to antagonize IFNAR and IFNGR signaling, no viral antagonists have been reported for IFNLR1 or IL-10R2. This raises the possibility that type III IFN signaling may be subject to different evolutionary pressures or specific resistance mechanisms against viral countermeasures. Future studies to determine whether viruses target these receptors indirectly or whether they are relatively protected parts of the IFN system could offer novel insights into host–virus co-evolution and anti-viral intervention strategies. Table 1 summarizes the strategies viral proteins employ to downregulate IFNRs.

## 5. Downregulation of JAKs

The JAK family is made up of four non-receptor tyrosine protein kinases: JAK1, JAK2, TYK2, and JAK3. Cytokine binding to their receptors activates JAK, which then relays downstream signaling. JAKs are composed of seven conserved regions, referred to as JAK homology (JH) domains. The JH1 domain serves as the active kinase and is responsible for phosphorylating target proteins. In contrast, the JH2 domain functions as a regulatory or pseudokinase region that controls JH1 activity and contributes to JAK-STAT interactions, in part by suppressing tyrosine kinase activity through its association with the kinase domain. The FERM domain, formed from portions of JH4 to JH7, and the SH2-like domain, derived from JH3 and part of JH4, are important for receptor binding. These regions help anchor JAKs to cytokine receptors by interacting with the membrane-proximal box1 and box2 motifs, enabling proper signal transduction [58].

JAKs are targeted by a variety of pathogens, including viruses, to ensure efficient virus replication. A study showed that IAV polymerase protein PB2 causes K48 ubiquitination and proteasomal degradation of JAK1 at lysine 859 and 860, which contributes to viral replication in mammalian cells and pathogenicity in a mouse model. PB2 from highly pathogenic avian influenza (HPAI) with the I283M and K526R mutations more effectively degrades mammalian JAK1, which enhances viral replication in mammalian cells [53]. ZIKV NS2B3 degrades JAK1 in a proteasomal manner, while reducing virus-induced apoptotic cell death [76]. The ASFV protein MGF505-7R antagonizes JAK-STAT signaling by targeting both JAK1 and JAK2 for degradation. MGF505-7R interacts with JAK1 and promotes its proteasomal degradation through the upregulation of the E3 ubiquitin ligase RNF125. In addition, MGF505-7R induces JAK2 degradation via proteasomal, lysosomal, and autophagic pathways by suppressing the expression of Hes5. Collectively, these mechanisms contribute to the inhibition of JAK-STAT signaling and facilitate viral immune evasion [77]. Foot and mouth disease virus (FMDV) VP3 abrogates IFN-γ-induced phosphorylation of STAT1 at Y701 and consequent downstream gene expression. Mechanistically, VP3 interacts with JAK1/2 and only degrades JAK1 via the lysosomal route [78]. ASFV MGF360-10L protein undergoes K48 ubiquitination and proteasomal degradation of JAK1, recruiting HECT and RLD domain-containing E3 ubiquitin protein ligase 5 (HERC5), for which residues K245 and K269 of JAK1 are critical for ubiquitination and degradation [79]. Swine acute diarrhoea syndrome coronavirus (SADS-CoV) nsp1 also degrades JAK1 via K11 and K48 ubiquitination and proteasomal degradation, inhibiting STAT1 phosphorylation, whereas F39 and K98 mutation of nsp1 abrogated the degradation [80]. The gammacoronavirus avian infectious bronchitis virus (IBV) C-terminal region of Nsp14 interacts with chicken JAK1 to degrade JAK1 in response to chicken IFN-γ via the autophagy pathway in HD11 chicken macrophages [81]. The small hydrophobic (SH) protein of human metapneumovirus (HMPV) has been found to promote the ubiquitination and subsequent proteasomal degradation of JAK1, thereby disrupting JAK-STAT signaling and dampening the host antiviral response [82]. A recent study has demonstrated that the N-terminal region (amino acids 1–152) of the Senecavirus A (SVA) 3D protein interacts with the FERM domain (amino acids 34–420) of JAK1. Mechanistically, SVA 3D recruits the E3 ubiquitin ligase RNF125, promoting K48-linked polyubiquitination of JAK1 at lysine residues K205 and K249. This modification targets JAK1 for proteasomal degradation, thereby suppressing downstream signaling. Furthermore, specific residues within the 3D protein E76, D90, D148, and D150 are critical for mediating the interaction between 3D and JAK1 [83]. The Measles virus (MV) PNT domain of the V protein interacts physically with the tyrosine kinase domain of JAK1 to inhibit STAT1 phosphorylation [84]. A predicted viral suppressor of cytokine signaling SOCS (vSOCS) with high homology to the vertebrate SOCS1, lacking a SOCS-box domain, is encoded by ORF103R of infectious spleen and kidney necrosis virus (ISKNV). This protein interacts with JAK1 and reduces its tyrosine kinase activity in vitro. ISKNV-vSOCS point mutations (F18D, S66A, S85A, and R64K) dramatically reduced the suppression of IFN-induced ISRE-promoter activation [85].

Human papillomavirus (HPV) E6 proteins have been shown to physically interact with TYK2, with a stronger association observed for HPV-18 E6 and a weaker one for HPV-11 E6. This interaction specifically involves the JH6-JH7 domains of TYK2, which are critical for its binding to the cytoplasmic region of IFNAR1 [86]. The NS5 protein of tick-borne encephalitis virus (TBEV) and Louping ill virus (LIV), which are related tick-borne flaviviruses, interfere with JAK-STAT signaling by interacting with the tyrosine kinase domain of TYK2. Furthermore, the RNA polymerase (RdRp) domain of tick-borne flavivirus NS5 is crucial for interacting with the TYK2 kinase domain, unlike the NS5 from mosquito-borne flaviviruses [87]. The EBV-encoded oncoprotein, latent membrane protein 1 (LMP-1), interacts with TYK2 and prevents TYK2 phosphorylation and inhibits IFN-α-stimulated STAT2 nuclear translocation and ISRE element transcriptional activity, whereas LMP-1 does not affect IFN-γ-induced GAS activation [54].

These viruses use multiple strategies to shut JAKs-mediated immune responses. The majority of proteins have evolved to degrade JAK1 using the ubiquitin-proteasome system in the host. JAK1 represents a critical signaling hub, as it is an essential component of type I, type II, and type III IFNR complexes; thus, its loss can broadly abrogate downstream IFN-driven antiviral responses. For example, IAV notably induces the production of type III IFNs [88], yet no viral protein has been reported to directly antagonize type III IFNRs. This apparent selectivity elucidates that virus immune evasion is not strictly receptor-centric but instead relies on convergence at shared downstream signaling nodes. In this context, degradation of JAK1 may indirectly suppress type III IFN signaling despite intact receptor engagement. Consistent with this multi-tiered strategy, IAV-HA promotes degradation of IFNAR1 and IFNGR1, thereby impairing type I and type II IFNs, leaving type III IFN receptors comparatively unaffected. Collectively, this pattern supports a model in which viruses, including IAV, prioritize the disruption of shared intracellular signaling hubs over complete receptor-level blockade of all IFN classes, potentially reflecting functional redundancy and tissue-specific roles of type III IFNs in epithelial barriers. Table 2 summarizes the tactics the virus proteins use to downregulate JAK proteins.

## 6. Downregulation of STATs

### 6.1. Downregulation of STAT1

STAT1, STAT2, STAT3, STAT4, STAT5a, STAT5b, and STAT6 make up the STAT family. Different STAT functions are regulated by six domains [58,89,90,91,92]. The transcription-activation domain (TAD), SH2 domain, linker domain, DNA-binding domain (DBD), coiled coil, and N-terminal domain (NTD) are present from the C-terminus to the N-terminus. When STAT1 is phosphorylated, it forms a dimer that penetrates the nucleus and activates the transcription of its targets. The NTD promotes the formation of STAT dimers, which enables their subsequent binding with transcription factors. The N-terminus can also increase interactions between STAT and transcription co-activators, the protein inhibitor of activated STAT (PIAS) family, and receptors, as well as influence nuclear translocation [93,94,95,96]. A potentially dynamic four-helix bundle makes up the coiled-coil domain that can interact with IRF9. This domain is associated with regulatory proteins and regulates nuclear import and export activities [97,98,99,100,101]. As the name implies, the linking domain connects the DBD to the SH2 domain structurally. It plays a role in STAT1 transcriptional regulation [100,101]. The SH2 domain is very conserved in the STAT family, which recognizes phosphotyrosine motifs of cytokine receptors and cooperates with activated JAK to drive the SH2 domain of STAT to interact with the tail of another STAT monomer after phosphorylation to form a homodimer or heterodimer [102,103,104]. TAD is essential for DNA transcription elements and the recruitment of co-activators via a conserved serine phosphorylation site, as well as transcriptional regulation. Cytokines, growth factors, G-protein-coupled receptors, non-receptor tyrosine kinases, or protein adaptors, such as MEKK1-C, can all activate STATs [105].

STAT1 is a key player in cellular response to IFNs [26]. Given the importance of STAT1 in the antiviral immune response, numerous viruses, including deadly ones like Ebola virus (EBOV) and SARS-CoV-2, have developed various ways to suppress this transcription factor. Extensive characterization has shown that most *Paramyxoviridae* viral proteins effectively block STAT1-mediated antiviral defense mechanisms. According to two similar studies, Simian virus 5 (SV5) or Parainfluenza virus 5 (PIV5) structural protein V degrades STAT1, not STAT2, via the proteasomal pathway induced by IFN-α/β and IFN-γ [106,107]. For this degradation of STAT1, the p127 subunit (DDB1) of the UV damage-specific DNA binding protein (DDB) is essential [108]. Another study confirmed that STAT1 is ubiquitinated by SV5 V protein in rabbit reticulocyte lysates [109]. The same study group proposed a dynamic scenario in which SV5 V functions as a bridge, bringing together a DDB1 and Cullin 4A-containing ubiquitin ligase complex and STAT1/STAT2 heterodimers, resulting in STAT1 degradation. The V protein of SV5 directly interacts with STAT2 and DDB1 [110]. The V protein of the Nipah virus (NiV), which is another type of paramyxovirus, suppresses IFN responses by forming large complexes with STAT1. It builds up in the cytoplasm through a Crm1-dependent process, alters the normal localization of STAT proteins, and blocks both their movement in response to IFN and the tyrosine phosphorylation of STAT1 [111]. Amino acids 174 to 192 of the NiV V protein have been identified as a Crm1-dependent nuclear export signal (NES). As IFN signaling is largely inhibited by the interaction between NiV V residues 100 to 160 and STAT1 residues 509 to 712, NES is not required for IFN signaling suppression. Whereas NES is required for STAT1 and STAT2 cytoplasmic retention as well as V protein shuttling [112]. The NiV V, W, and phosphoprotein (P) proteins are all encoded by the same viral gene and contain the same N-terminal region with 407 amino acids but different C-terminal sequences. The N-terminal domain of 50 to 150, which is common for all three proteins, interacts with STAT1 and inhibits IFN responses. Although the NiV V and P proteins share a STAT1-binding domain, they keep STAT1 trapped in the cytoplasm. In contrast, the W protein holds STAT1 in the nucleus. Together, these actions block STAT1 function in both the cytoplasm and the nucleus [113]. A recent study showed that NiV P sequestration of STAT1 into viral inclusion bodies (IBs) that inhibit STAT1 phosphorylation and nuclear translocation [114]. The Sendai virus (SeV) C protein physically interacts with both unphosphorylated and phosphorylated STAT1, creating large molecular weight complexes from STAT1 phosphorylated homodimers [55]. Later, the same study group discovered that the SeV C protein inhibits STAT1 phosphorylation and dephosphorylation without impairing STAT1 nuclear translocation upon IFN stimulation [115]. Moreover, SeV all four C proteins (C′, C, Y1 & Y2) physically interact with STAT1, but only the larger forms degrade STAT1 via a mono-ubiquitination proteasomal system. Furthermore, the inability of a null mutant (C^F170S^) to bind STAT1 implies that this interaction is physiologically important [116]. Moreover, the C-terminal half-fragment (D1; aa 85–204) of the C protein of the SeV inhibits in vitro binding of the NTD of phosphorylated-STAT1 dimer to the GAS probe and IFN-γ response as D1 interacts with NTD of STAT1 [117]. Hendra virus (HeV) V protein, closely related to NiV V protein with 58% shared identity, inhibits IFN signaling, interacting with STAT1 [118]. Neurovirulent canine distemper virus (CDV) A75/17 strain N-terminal region of non-structural (NS) protein V specifically Y110 within N-terminal interacts with STAT1, disrupting type I IFN-induced signaling [119]. Notably, the mumps virus (MuV) V protein ubiquitinates and degrades STAT1. The C-terminal region of STAT1 is not essential for V protein interaction for its degradation. But C189, C207, and C214 cysteine residues of the V protein are important for STAT1 degradation. C189A and C207A mutations of the V protein disrupt the STAT1-V protein interaction [120]. Another study suggested that the MuV V protein functions to degrade STAT1 incompletely and inhibit the Y701-STAT1 phosphorylation completely. But C189A, C207A, and C214A mutations make the V protein non-functional, restoring STAT1 degradation and Y701-STAT1 phosphorylation [121]. Measles virus (MV), another paramyxovirus, encodes three proteins from its P gene: the P protein (an essential cofactor for the RNA polymerase), along with C and V proteins that perform multiple functions such as immune evasion. A study suggested that the Y110 amino acid of the P protein of MV is required to suppress phosphorylation and nuclear translocation of STAT1 [122]. Another study demonstrated that both the N- and C-terminal regions of the MV-V protein (PNT and VCT) play roles in suppressing IFN-α/β signaling. The study also confirmed a direct interaction between MV-V and STAT1, which leads to reduced STAT1 phosphorylation. Importantly, the Y110H mutation weakened its ability to bind STAT1 and impaired its capacity to inhibit STAT1 phosphorylation [84]. The C protein of the human parainfluenza virus type 1 (HPIV1) interacts with both phosphorylated and unphosphorylated STAT1, inducing an antiviral state in the cell. But the F170S mutation of the C protein impairs the interaction with STAT1 HPIV1. C proteins form a stable complex with STAT1 in perinuclear granules, preventing STAT1 translocation to the nucleus [123]. Newcastle disease virus (NDV), a member of the genus *Avulavirus* within the family *Paramyxoviridae*, encodes a V protein that degrades phosphorylated STAT1 through the proteasome pathway to block IFN-α signaling [124].

*Coronaviridae* family viruses also have shown multiple strategies to avoid IFN-induced antiviral immunity. For example, SARS-CoV-2 N protein amino acids 319–422 are indispensable for the interaction with STAT1 to block STAT1-JAK1 association, inhibiting phosphorylation of STAT1 [125]. SARS-CoV-2 helicase NSP13 was shown to interact with STAT1 with a high-affinity purification assay (S-pulldown) coupled with quantitative mass spectrometry (MS) to identify the cellular interactome of NSP13. Further analysis of truncation mutations revealed that the NSP13 protein 1A domain is vital for the interaction with STAT1 [126]. Moreover, SARS-CoV-2 NSP13 interacts with STAT1 to prevent it from being phosphorylated by JAK1 kinase. The nucleic acid-binding-deficient mutants K345A, K347A, and the NTPase-deficient mutant E375A of NSP13, both of which are necessary for helicase activity, lose their capacity to decrease STAT1 phosphorylation [127]. Also, the SARS-CoV-2 spike (S) protein is associated with STAT1 to block the STAT1-JAK1 interaction, subsequently suppressing STAT1 phosphorylation. The S1 subunit of the S protein contains the receptor-binding domain (RBD), which is responsible for binding to the host cell receptor ACE2 and interacts with STAT1 [128]. Another study demonstrated that the highly conserved S2 domain of the SARS-CoV-2 S protein interacts with STAT1, disrupting the phosphorylation, impairing both the assembly and nuclear translocation of the ISGF3 complex [129]. SARS-CoV-2 main protease (Mpro, also called 3CLpro or nsp5) binds with STAT1 and degrades via the autophagy pathway, downregulating the IFN signaling pathway. Mechanistically, nsp5 enhances the interaction between STAT1 and autophagic receptor p62, whereas enzymatic inactive mutant C145S of nsp5 reduces p62-STAT1 interaction and restores STAT1 degradation [130]. Moreover, SARS-CoV-2 ORF6 interacts directly with STAT1 via its C-terminal to impair the IFN signaling [131]. Furthermore, the nsp7 of the porcine epidemic diarrhoea virus (PEDV) binds with the DBD of STAT1, which sequesters the interaction between Karyopherin α1 (KPNA1) and STAT1, thereby limiting ISGF3 nuclear transport. The third α-helical region of nsp7 is crucial for the interaction with STAT1 [132]. The NS7a protein of porcine delta coronavirus (PDCoV) impedes STAT1 phosphorylation without interacting with it and the nuclear accumulation of ISGF3. Notably, the L4 residue of the GX2021-1 strain is vital for the IFN antagonism [133]. Another protein of PDCoV nucleocapsid (N) protein binds to STAT1 and inhibits its nuclear translocation, a function mediated by its C-terminal domain, thereby blocking the IFN signaling pathway [134].

Several viruses that belong to the *Picornaviridae* family have been shown to counteract the IFN signaling pathway. For instance, the FMDV Lb leader protease (Lbpro) directly interacts with and cleaves STAT1, inhibiting IFN-β-induced signaling, whereas the catalytically inactive mutation C51A of Lbpro fails to cleave STAT1. The study predicted that the cleavage site of STAT1 is between aa 252–502 [135]. Notably, EV71 3D reduces tyrosine phosphorylation (Y701) of STAT1, reducing STAT1 mRNA expression, while 2A protein reduces STAT1 serine phosphorylation (S727) to inhibit IFN-γ-induced antiviral gene transcription [136]. The 3C protease of respiratory enterovirus D68 (EV-D68) inhibits IFN signaling by cleaving STAT1 at the Q131 residue through its cysteine protease activity. In contrast, the 3C proteases from EV-A71, coxsackievirus A16, and echoviruses do not exhibit this activity [137]. Seneca Valley Virus (SVV) 3C^pro^ degrades STAT1 via its protease activity, which is dependent on the caspase pathway, suppressing JAK-STAT signaling [40]. A similar study suggested that SVV 3C^pro^ exerts its protease activity by selectively cleaving and degrading STAT1, thereby blocking the formation and nuclear translocation of the ISGF3 complex. In both human and porcine STAT1, cleavage occurs at the L693/D694 motif [41].

Several *Herpesviridae* family viruses have been shown to target STAT1 as a mechanism for evading host antiviral immunity. For example, the ICP22 protein of HSV-2, identified as an E3 ligase, interacts with and ubiquitinates STAT1, followed by degradation via the proteasomal pathway [138]. Bovine alpha herpesvirus 1 (BoHV-1) major tegument protein, VP8, interacts with cellular STAT1 to inhibit IFN-β signaling. Two VP8 domains, amino acids 259- 482 and 632–686, were discovered to be responsible for the interaction with STAT1 [139]. Another BoHV-1 tegument protein, UL41, cleaves the mRNA of STAT1 to inhibit STAT1 translation, repressing the activity of IFN response elements and the generation of ISGs [45].

HCV belongs to *Flaviviridae* and expresses full-length HCV polyprotein, HCV structural protein (HCV core, E1, and E2 proteins), and NS3-4A, which are associated with proteasome-dependent degradation of STAT1 and phosphorylated STAT1. Only HCV core, but not HCV E1, E2, NS3, NS4, or NS5, binds to STAT1. HCV structural protein, NS3-4A, and NS4B, but not NS5A, were able to block ISRE activity induced by IFNs [140]. Here, the N-terminal portion of HCV core protein (aa 1–23) interacts with the C-terminal region of STAT1, specifically the SH2 domain (aa 577–684) that is critical for STAT1 hetero- or homodimerization [141]. Conversely, another study group identified that HCV NS5A protein interacts with the N-terminal (1–488) of STAT1 and inhibits STAT1 phosphorylation in hepatocyte-derived cell lines [142]. Moreover, HCV genotype 1- NS5A protein C-terminal interacts with STAT1 to reduce the IFN action [143]. ZIKV Brazil/2016/INMI1 NS4B protein shared homology with the IFN inhibitory domain of DENV2 NS4B, blocks STAT1 phosphorylation, and consequently blocks the nuclear transport [144]. Later, the same study group found that ZIKV NS2A degrades STAT1 via the proteasomal pathway. NS2A aa 12–100, conserved among other flaviviruses, is important for IFN antagonism [145].

Rabies virus (RABV) (which belongs to the family *Rhabdoviridae*), P protein, a cofactor of RNA polymerase, C-terminal part (aa 268–297) interacts with a region containing the DBD and the coiled-coil domain of phosphorylated STAT1. P protein does not inhibit STAT1 phosphorylation or degradation, but it does prevent IFN-induced STAT1 nuclear accumulation [56]. Later, the P protein is shown to bind with phosphorylated STAT1 to keep it in the cytoplasm. Lacking ten C-terminal residues (288–297) of P protein does not show an inhibitory effect on either GAS- or ISRE-driven reporter gene expression and nuclear accumulation of STAT1 [146]. Furthermore, the P protein of RABV is shown as a nucleocytoplasmic shuttling protein (including NES and NLS), and STAT1 localization is associated with P localization. In cells expressing a nuclear form of P, STAT1 is retained in the nucleus, whereas in cells expressing a cytoplasmic version of P, STAT1 is retained in the cytoplasm. Nuclear version of P blocks the IFN-induced DNA binding of phosphorylated STAT1. Therefore, the RABV P protein can inhibit IFN signaling by sequestering STAT1 in both cytoplasmic and nuclear compartments [147].

Large DNA viruses, including ASFV and vaccinia virus (VV), have evolved multiple strategies to diminish IFN signaling. MGF360-9L from ASFV interacts with STAT1 and degrades via the apoptosis pathway [148]. Also, ASFV A104R, a histone-like protein, inhibits STAT1 phosphorylation without interaction, and mutations of A104R (R/H69,72D and K/R92,94,97E) fail to inhibit STAT1 phosphorylation, elucidating that the DNA-binding capacity of A104R is indispensable to block IFN signaling [149]. ASFV F778R protein inhibits IFN signaling by weakening the nuclear accumulation of activated STAT1 [150]. A recent study demonstrated that the ASFV protein pI7L inhibits the IFN-γ-induced JAK-STAT signaling pathway. Mechanistically, pI7L competes with JAK1 for binding to the SH2 domain of STAT1, thereby suppressing STAT1 phosphorylation and homodimerization, ultimately blocking its nuclear translocation and reducing the expression of IFN-γ-stimulated genes. Notably, pI7L undergoes tyrosine phosphorylation at residue Y98, which is essential for its interaction with STAT1 and the resulting inhibitory effect on STAT1 activation [151]. Another ASFV protein, MGF505-4R, has been shown to interact with STAT1, thereby disrupting the assembly of the ISGF3 heterotrimer. This interference inhibits the nuclear translocation of pSTAT1 and subsequently suppresses the transcription of ISGs [152]. The VV phosphatase (VH1), sensitive to the phosphatase inhibitor Na_3_VO_4_ but not to okadaic acid, binds and dephosphorylates STAT1, blocking IFN-γ signaling [153].

HBV polymerase protein also interferes with STAT1 nuclear translocation [154]. The same study group identified that HBV polymerase associates with protein kinase C-δ (PKC-δ) and perturbed PKC-δ phosphorylation and its association with STAT1, which resulted in the suppression of STAT1 S727 phosphorylation for the inhibition of IFN-α signaling in human hepatic cell lines [155]. Also, the EBOV VP24 was shown to associate with purified STAT1 of the 1–683 aa region. Crystal structures and accompanying biochemical analysis of the EBOV VP24 showed that VP24 interacts with STAT1 directly. For the first time, the crystal structures of VP24 are obtained from pathogenic (Sudan) and nonpathogenic to humans (Reston) EBOV for the study [156]. The N-terminal 1–220 amino acid residues of EBOV VP35 inhibit type I IFN signaling by blocking the phosphorylation of endogenous STAT1 and suppressing its nuclear translocation [157]. Severe fever with thrombocytopenia syndrome virus (SFTSV) nonstructural protein (NSs) interacts with STAT1 and suppresses phosphorylation at S727, thereby inhibiting the activation of STAT1 [158]. In contrast, Rotavirus (RV) NSP1 inhibits the IFN signaling pathway by targeting Y701-STAT1 phosphorylation, and the RING finger motif of NSP1 is not necessary for this inhibitory function [159]. Chikungunya virus (CHIKV) belongs to the family *Togaviridae* and is an arthropod-borne virus (arbovirus). The CHIKV nsP2 C-terminal methyltransferase-like domain specifically inhibits the IFN response by promoting the Crm1-mediated nuclear export of pSTAT1. Though nsP2 is present in both the cytoplasm and the nucleus in infected cells, the KR649AA mutations localize exclusively to the cytoplasm and no longer specifically inhibit JAK/STAT signaling and nuclear translocation of STAT1 [160]. A study showed that the expression of Respiratory Syncytial Virus (RSV)-NS1 protein is able to increase IFN-α-induced STAT1 phosphorylation; however, it simultaneously suppresses the activity of ISRE and GAS promoters. Mechanistically, RSV-NS1 binds to STAT1, hindering its nuclear translocation. This effect is further supported by a reduced interaction between STAT1 and the nuclear transport adapter KPNA1, indicating that RSV-NS1 impairs STAT1 signaling by blocking its nuclear import [161]. Similarly, the HPV E1 protein interacts with STAT1, impairing the assembly of the ISGF3 complex and obstructing the nuclear translocation of STAT1, STAT2, and IRF9. This disruption ultimately results in the suppression of ISGs [57]. Table 3 provides an overview of the strategies employed by virus proteins to downregulate STAT1.

### 6.2. Downregulation of STAT2

The STAT family member with the highest structural and functional divergence is STAT2, which has only been discovered to be activated by IFN-I and IFN-III. There are 24 exons in the human and mouse STAT2 gene. The human STAT2 protein is the longest and largest of the STATs due to its amino acid composition and 113 kDa molecular weight. The amino acid sequences of mouse and human STAT2 proteins, in contrast to other STATs, are very different, particularly in the C-terminal regions [162]. Upon cytokine activation, STAT proteins typically form homodimers. However, STAT2 only forms heterodimers with simultaneously active STAT1, which then associates with another DNA-binding protein, IRF9, to produce the transcription factor ISGF3 [163]. The generation of unphosphorylated STAT dimers is mediated by the NTD of STATs, which has eight short helices and covers the first 130 amino acids (aa) of the protein. A coiled-coil domain (aa 135–315) that has a superhelix structure made of four -helices wrapped around one another follows this section, necessary for IRF9 interaction [164].

Many viruses in the *Paramyxoviridae* family target STAT2 to inhibit IFN signaling. In particular, by building high-molecular-weight complexes with STAT2, the NiV V protein suppresses IFN responses. The study found that the NiV V protein builds up in the cytoplasm through a Crm1-dependent process. In doing so, it alters the normal localization of STAT proteins within the cell and blocks their usual redistribution in response to IFN stimulation [111]. The NiV V protein amino acids 100 and 300 interact with STAT2; nevertheless, deletion of residues 230 to 237 drastically reduced STAT2 co-precipitation. Furthermore, interactions between V protein and cellular STAT1 are required for STAT2 binding, and consecutive immunoprecipitations show that V, STAT1, and STAT2 can form a tripartite complex [112]. hPIV2 V protein expressing human 2fTGH cells degrade STAT2 and proteasomal inhibitor, MG132 restores the STAT2 degradation [107]. Moreover, the same study group using programmed rabbit reticulocyte lysates and bacterially expressed and purified hPIV2 V protein demonstrated that it polyubiquitinates and degrades in vitro-translated STAT2. The degradation of STAT2 is more prominent in human cells than in animal cells [109]. The trp motif (W-(X)3-W-(X)9-W) of hPIV2 V protein associates with and degrades STAT2 whereas C193/197A, C209/211/214A, C218/221A, F143S, and F207E mutations of V protein lose the ability to degrade STAT2 [165]. HeV V protein inhibits cellular responses to IFN through binding and cytoplasmic sequestration of STAT2, but not STAT3 [118]. MuV-V protein inhibits the Y689-STAT2 phosphorylation, and conserved C-terminal cysteine-rich region mutations (C189A, C207A, and C214A) restore STAT2 phosphorylation [121]. In particular, the extensively conserved C-terminal zinc finger domain of the MV V protein binds STAT2 and impairs IFN-α/β signal transduction. The contact surface for STAT2 association, defined by molecular modelling and mutagenesis, shows that aspartic acid residue D248 is important for STAT2 association [166]. The V protein of neurovirulent CDV A75/17 strain interacts with STAT2, inhibiting its nuclear translocation. Single mutation at position 110 within the VNT domain of CDV V protein, resulting in a mutant that lost STAT2 association partially, whereas both the VNT and VCT are required for STAT2 association [119].

The *Coronaviridae* family has evolved strategies to evade IFN signaling by specifically targeting STAT2. Likewise, SARS-CoV-2 N protein plays a role in inhibiting ISG transcription. The amino acids 1–361 of N interact with STAT2 to block STAT2-TYK2 interaction, inhibiting phosphorylation of STAT2 [125]. Moreover, SARS-CoV-2 ORF7a protein inhibits STAT2 phosphorylation, blocking ISG transcription [167]. Another SARS-CoV-2 protein, NSP1, shuts off STAT2 translation, reducing ISRE activation, whereas KH164AA, which is reported as unable to inhibit host cell translation, restores those processes [47]. The S2 domain of the SARS-CoV-2 S protein interacts with STAT2, disrupting its phosphorylation, impairing both the assembly and nuclear translocation of the ISGF3 complex [129]. The NS7a protein of PDCoV associates with STAT2-DBD and STAT2-SH2 domains, inhibiting STAT2 phosphorylation, hindering the formation and nuclear accumulation of ISGF3 [133]. The E protein of SADS-CoV, a bat coronavirus closely related to *Rhinolophus* bat coronavirus HKU2, promotes STAT2 degradation through the autophagy pathway. This process depends on E protein ubiquitination, while K61 is essential for maintaining E protein stability but does not participate in ubiquitination. Mechanistically, the E protein facilitates immune evasion by inducing STAT2 autophagic degradation via the NBR1 autophagy cargo receptor and optineurin (OPTN) receptors [39].

Flavivirus NS5 proteins depend heavily on human STAT2 degradation as a key mechanism for suppressing IFN signaling during host immune evasion; for example, the DENV NS5 polymerase ubiquitinates and degrades STAT2 via the proteasomal pathway. Deletion of the first 10 amino acids of NS5 prevents STAT2 degradation while still interacting with STAT2, which is inhibited by the deletion of aa 278–900 of NS5. Although both unprocessed and processed NS5 bind STAT2, only proteolytically processed NS5 can efficiently facilitate STAT2 degradation [168]. Later, it was found that the DENV-NS5 protein recruits ubiquitin-protein ligase E3 component n-recognin 4 (UBR4) to STAT2 for its degradation [169]. YFV NS5 exclusively interacts with STAT2 induced by IFNs, whereas the K6R mutation of NS5 disrupts STAT2 interaction. For STAT2 interaction, NS5 must be K63-linked ubiquitinated by E3 ligase TRIM23 at K6 residue of NS5 [170]. NS5 of ZIKV interacts with human STAT2, not mouse STAT2, to counteract the IFN pathway [171]. Later, a genome-wide CRISPR/Cas9 screen identified ZSWIM8 as the substrate receptor of the Cullin3-RING E3 ligase complex, which is essential for NS5-induced ubiquitination and subsequent degradation of STAT2. The N-terminal ZSWIM8-Box domain is critical for its association with CUL3, without disrupting its interaction with the ZIKV NS5 protein [172]. Noticeably, another ZIKV protein, NS2A, was demonstrated to degrade STAT2 via the proteasomal pathway, disrupting IFN signaling [145]. WNV Kunjin (KUN) strain MRM61C nonstructural proteins NS2A, NS2B3, NS4A, and NS4B, but not NS1 and NS5 inhibit STAT2 translocation to the nucleus [173].

Picornaviruses utilize diverse mechanisms to overcome IFN signaling and evade host antiviral responses. Such as FMDV Lbpro directly interacts with and cleaves STAT2 (site spanning residues 140–150 aa (QQHEIESRIL) via its protease activity [135]. FMDV 3D polymerase, an RNA-dependent RNA polymerase (RdRp), interacts with STAT2, thereby suppressing its phosphorylation and subsequent nuclear translocation, thereby impeding the activation of the IFN signaling pathway [174]. The 3C^pro^ of SVV or SVA cleaves and degrades STAT2 through its protease activity, which relies on the caspase pathway, thereby suppressing JAK-STAT signaling. 3C^pro^ was further found to cleave STAT2 at glutamine 758 (Q758) within the TAD; this cleavage attenuated STAT2’s ability to activate ISRE activity and induce ISG production [40]. A similar study suggested that STAT2 has two cleavage sites. In STAT2, a shared cleavage site at Q707 is present in both human and porcine, while the second site varies: residues 754–757 (V-L-Q-S) in human STAT2, and Q758 in porcine STAT2 [41].

SFTSV and Heartland virus (HRTV), members of the *Phenuiviridae* family (a group of arthropod-borne RNA viruses), have evolved strategies to evade IFN signaling. SFTSV NSs interacts with STAT2, and the DBD of STAT2 is vital for NSs-STAT2 interaction. NSs sequesters STAT2 in viral IBs, impairing IFN-induced STAT2 phosphorylation and nuclear translocation [175]. HRTV NS protein (HNSs) interacts with the DNA-binding and linker domains of STAT2, inhibiting phosphorylation only of STAT2, resulting in the inhibition of IFN-α- and IFN-λ-mediated antiviral responses [176].

STAT2 is directly interacted with, ubiquitinated by, and degraded by HSV-2 ICP22, through the proteasomal route [138]. Furthermore, the EBV tegument protein BGLF2 interacts with STAT2 and is K48 polyubiquitinated and degraded by the proteasome. BGLF2 connects with cullin 1 E3 ubiquitin ligase to enhance recruitment of STAT2 for degradation [177]. The acidic domain of human cytomegalovirus (HCMV) immediate-early 1 (IE1) protein interacts with STAT2. Specifically, SUMOylation within the acidic domain of IE1 negatively affects its binding to STAT2, thereby abolishing its ability to repress IFN-regulated gene expression [178].

The ASFV cysteine protease core domain of pS273R interacts with NTD of STAT2, reducing IFN signaling. pS273R recruits the E3 ubiquitin ligase DCST1, resulting in K48-linked polyubiquitination at K55 of STAT2 and subsequent proteasome-dependent degradation of STAT2 independent of its protease activity [179]. pI215L, an ASFV ubiquitin-conjugating enzyme, binds with, ubiquitinates, and degrades STAT2 via the proteasomal pathway using its catalytic activity [180]. MGF360-9L from ASFV interacts with STAT2 and degrades via the ubiquitin-proteasome pathway [148]. ASFV pB475L binds to the CTD of STAT2, disrupting the heterodimerization of STAT1 and STAT2 and thereby preventing their nuclear translocation. Molecular docking analysis revealed that specific residues H36, S79, L82, E268, G373, E456, and S458 of pB475L are critical for its interaction with STAT2 [181]. Another ASFV protein, MGF505-4R, has been shown to interact with STAT2, thereby disrupting the assembly of the ISGF3 heterotrimer. This interference inhibits the nuclear translocation of pSTAT2 and subsequently suppresses the transcription of ISGs [152]. The Monkeypox virus (MPXV) poxin-schlafen (PoxS) fusion protein binds to STAT2 and retains it in the cytoplasm, thereby preventing its nuclear translocation. Effective sequestration of STAT2 requires both the schlafen domain and the active site of the 2′3′-cGAMP nuclease. This dual functionality is essential for the suppression of ISG expression, highlighting a multifaceted mechanism by which MPXV evades host immune responses [182]. VV C6 is associated with the TAD of STAT2 to inhibit ISRE-induced ISG gene transcription [183].

NTD of (aa 57 to 59) nsp11 of PRRSV interacts with STAT2-NTD and the coiled-coil domain and degrades STAT2 via K48-linked ubiquitination. The nsp11 amino acid K59 is indispensable for STAT2 degradation [184]. The capsid protein (Cap) of porcine circovirus 3 (PCV3) interacts with the TAD of STAT2 but has no effect on STAT2 expression or phosphorylation levels, nor does it disrupt the heterodimerization of pSTAT1 and pSTAT2 though it inhibits type I IFN signaling [185]. Bluetongue virus (BTV) NS3 interacts with and degrades STAT2 via the autophagic/lysosomal pathway, inhibiting IFN signaling. The study showed that NS3 undergoes K63 polyubiquitination at K13 and K15 and recruits an E3 ligase for STAT2 degradation [186].

Recent studies have demonstrated that the HPV16 E4 protein acts as a negative regulator of the JAK-STAT signaling pathway, resulting in diminished expression of ISGs. Mechanistically, E4 interacts with STAT2, thereby disrupting the assembly of the ISGF3 complex and inhibiting the nuclear translocation of STAT1, STAT2, and IRF9 [187]. RABV protein P links only with IFN-α or IFN-γ induced phosphorylated STAT2, not with inactivated STAT2, to keep it in the cytoplasm. Lacking ten C-terminal residues (288–297) of P protein loses the ability to keep STAT2 in the cytoplasm [146]. RSV NS1 and NS2 proteins degrade STAT2 via the proteasomal pathway. NS1 has elongin C and cullin 2 binding consensus sequences that interact with elongin C and cullin 2 in vitro. Thus, NS1 functions as an E3 ligase to ubiquitinate and degrade STAT2 in the proteasomal pathway [188].

The majority of viral proteins that target STATs act on either STAT1, STAT2, or both proteins. Similar to JAK1, STAT1 is also one of the central hubs downstream of JAKs to activate the antiviral cascade in response to all types of IFNS: I, II, and III. Some virus proteins degrade STATs, and others inhibit complex formation and phosphorylation. Even though the Y701 phosphorylation of STAT1 induced by IFN or other cytokines is generally considered an essential step in the canonical activation of the JAK/STAT pathway, it can also be phosphorylated on S727 in the C-terminal TAD [189]. However, without tyrosine phosphorylation, STAT1 has been shown to enhance antiviral gene expression through non-canonical activation [190]. Furthermore, unphosphorylated -STAT1 and -STAT2, together with -IRF9, can assemble into a non-canonical complex known as U-ISGF3. This complex forms under conditions of high IRF9/STAT1/STAT2 expression and can be induced by low levels of IFN-β, even in the absence of tyrosine phosphorylation (U-ISGF3) [19,191]. Furthermore, U-ISGF3 contributes to the constitutive expression of ISGs under homeostatic conditions, establishing a basal antiviral defense against viral infections [192,193]. Moreover, IKKϵ-mediated STAT1 S708 phosphorylation is crucial for IFIT2 expression to control WNV [194]. All this evidence suggests that STAT1 may be activated without IFNs and activated upstream, making STAT1 a significant target in IFN signaling, which is targeted by more virus proteins. This event further emphasizes that degradation of STAT1 is a more promising immune evasion strategy than that of phosphorylation inhibition deployed by viruses. On the other hand, most of the virus proteins that degrade or block the function of STAT1 also interact and degrade STAT2. Loss of STAT1 does not completely abolish type I IFN signaling, as STAT2 can form homodimers that interact with IRF9 to initiate an alternative transcriptional program. Although delayed compared with canonical ISGF3 signaling, this pathway promotes prolonged ISG expression and antiviral activity [162,195]. Therefore, viruses may target both STAT1 and STAT2 for degradation to prolong the suppression of antiviral immunity for efficient replication. Table 4 outlines the diverse strategies reported for STAT2 downregulation by virus proteins.

## 7. Downregulation of IRF9

IRF9 is best characterized as a transcription factor that regulates the expression of ISGs downstream to mediate the type I IFN response (as a component of ISGF3) [196,197]. IRF9 is composed of DBD and the IRF-associated domain (IAD) that are connected by a linker. Following the IFN stimulation, IRF9 forms the ISGF3 complex with STAT1 and STAT2 instead of homodimers. The ISRE consensus sequence 5-A/GNGAAANNGAAACT-3′ at the promoter region of ISGs is recognized by DBDs of IRF9 and STAT1, while STAT2-DBD interacts with non-consensus sequences within the ISGF3 complex [197]. IRF9 has no transcriptional activity on its own and has a low affinity for ISREs. STAT1 and STAT2 are essential for effective DNA binding and transcriptional activity, which is associated with variable DNA sequence selectivity [197,198]. IAD of IRF9 oversees binding to STAT2 coiled-coil domain. IRF9-IAD lacks the autoinhibitory region, which explains prior theories that activation by signal-induced phosphorylation is not required for IRF9-STAT2 connection [197,199]. However, an early investigation revealed that IRF9 might be phosphorylated constitutively inside the DBD without IFN stimulation [200,201].

Several viral proteins have been implicated in evading type I and III IFN responses by degrading the IRF9 protein. ORF63 of the alphaherpesvirus Simian varicella virus induces proteasomal degradation of IRF9. Likewise, ORF63 of varicella-zoster virus (VZV) similarly degrades IRF9 to inhibit ISGs induction [202]. ASFV pI215L degrades IRF9 via autophagy irrespective of its ubiquitin-conjugating activity [203]. ASFV MGF360-12L degrades IRF9, thus subverting IFN signaling [204]. HSV-2 ICP22 interacts directly with and ubiquitinates and degrades IRF9 via the proteasomal pathway to inhibit IFN signaling [138]. RV NSP1 degrades IRF9 [205], and EV71 3C^pro^ cleaves IRF9 in vitro [206]. SVV 3C^pro^ degrades IRF9 through its protease activity, which relies on the caspase pathway, thus perturbing JAK-STAT signaling [40]. Porcine bocavirus (PBoV) NP1 negatively regulates the IFN signaling pathway, blocking the ISGF3 DNA-binding activity by targeting the DBD of IRF9 [207]. PRRSV nsp11, a nidovirus-specific endoribonuclease (NendoU), interacts with the IAD domain of IRF9 and impairs the formation and nuclear translocation of ISGF3 with NendoU activity-independent mechanism [208]. The NS7a protein of PDCoV associates with IRF9, impeding the formation and nuclear accumulation of ISGF3. Notably, the L4P mutation of PDCoV led to disrupted IFN signaling pathway, reducing the interaction with IRF9 [133]. The S2 domain of the S protein of SARS-CoV-2 interacts with IRF9, impairing both the assembly and nuclear translocation of the ISGF3 complex. While the S protein binds to IRF9, thereby preventing its recruitment by STAT2. This interference is mediated through competition with the STAT2-CCD for IRF9 binding, and the mutation of residues L233, R236, L274, and F283 to alanine within IRF9-IAD eliminated the interaction [129]. Recent evidence suggests that the HPV E4 protein can inhibit JAK-STAT signaling, leading to a decrease in the expression of ISGs. E4 interacts with IRF9, which disrupts the formation of the ISGF3 complex and prevents STAT1, STAT2, and IRF9 from translocating into the nucleus [187]. Mildly myocarditic reovirus type 1 Lang (T1L) M1 gene product, μ2 protein, induces accumulation of IRF9 in the nucleus of reovirus-infected cells, repressing the IFN signaling pathway [209]. Also, the PRV C-terminal region of early protein 0 (EP0) inhibits IRF9 transcription [44].

Targeting IRF9 is a highly effective immune evasion strategy since IRF9 serves as the essential DNA-binding component of the ISGF3 complex, which mediates the expression of type I and type III ISGs. In the absence of IRF9, ISGF3 may not efficiently bind ISREs within gene promoters, resulting in a profound reduction in ISG transcription and antiviral defense. Moreover, IRF9 functions as a core hub that is required not only for canonical ISGF3 (STAT1-STAT2-IRF9) activity but also for non-canonical STAT2-IRF9 and U-ISGF3 pathways. Consequently, viral proteins that disrupt IRF9 function can simultaneously disable multiple IFN-dependent antiviral mechanisms, creating a critical bottleneck in host innate immunity and facilitating efficient viral replication. Table 5 summarizes the mechanisms of virus proteins used to downregulate IRF9.

## 8. Downregulation of KPNA

Importin α, also known as KPNA, was discovered as an adapter protein that connects classical NLS-containing proteins to importin-β, also known as karyopherin β (KPNB). This protein has three important structural domains: an N-terminal importin-β binding (IBB) domain, armadillo (Arm) repeats that serve as internal cargo classical NLS-binding sites, and a C-terminal region that binds to the nuclear export component of importin [210]. For instance, there are three subfamilies of human importin α: subfamily 1α (importin α5 (KPNA1), α6 (KPNA5), and α7 (KPNA6); subfamily α2 (importin α1 (KPNA2) and α8 (KPNA7); and subfamily α3 (importin α3 (KPNA4) and α4 (KPNA3). Studies have demonstrated that activated STAT1 interacts with members of the subfamily 1α KPNA [210,211]. For cargo proteins to be nuclear localized, it has been established that KPNAs interact with KPNB [212].

Although no host proteins have been identified as regulators of KPNAs, many viral proteins are recognized to negatively regulate KPNAs. Most of these viral proteins physically interact with KPNAs to block their function, while some degrade the protein. For example, PRRSV Nsp1β ubiquitinates and degrades KPNA1 via the proteasomal pathway, blocking IFN signaling. Valine at position 19 of Nsp1β is critical for the degradation of KPNA1 [213]. FMDV 3C^pro^ causes proteasome- and caspase-independent protein degradation of KPNA1, inhibiting STAT nuclear translocation and ISRE and GAS promoter activity [214,215]. With its protease activity, EV-A71 2B degrades KPNA1 via its putative hydrophilic domain (H1) in the N-terminus of 2B, which is characterized as critical for the release of cytochrome c into the cytosol for the activation of pro-caspase-3. 2B reduces the formation of p-STAT1/KPNA1, which leads to the inhibition of IFN signaling [216]. E6 protein of HPV ubiquitinates and degrades KPNA1 with host E6AP protein, an E3 ligase. The E6 protein with E6AP degrades KPNA1, leading to the suppression of nuclear transport of IFN-γ-induced phosphorylated STAT1 in cervical cancer cells [217]. SVV 3C^pro^ degrades KPNA1 via its protease activity, which is dependent on the caspase pathway, suppressing JAK-STAT signaling [40]. PDCoV N protein impairs STAT1 signaling by promoting the lysosome-dependent degradation of KPNA2, thereby disrupting downstream activation in the JAK-STAT signaling pathway [134].

EBOV VP24 interacts with KPNA1, KPNA5, and KPNA6, the NLS receptor for phosphorylated STAT1, and blocks KPNA-pSTAT1 interaction. Correspondingly, KPNA1 interacts with VP24 with its C-terminal domain, where in the pSTAT1 binding region. Notably, ARM10 of KPNA5 is vital for the interaction with VP24 [211,218,219]. SARS-CoV-2 ORF6 induces the cytoplasmic localization of KPNA2 compared to KPNA1, which is less. ORF6 interacts directly with KPNA1 with low affinity (low when IFN is untreated and high with IFN stimulation) and KPNA2 with higher affinity (with or without IFN stimulation). However, the study suggests that while ORF6 causes a modest disruption of the general importin α/β1-dependent nuclear transport pathway, its main effect is the direct and selective blockade of STAT1 entry into the nucleus. This prevents proper IFN signaling and ultimately helps the virus replicate more efficiently [131]. HBV polymerase interferes with the nuclear transport of STAT1/2 by competitively binding to the area of KPNA1 necessary for STAT1/2 recruitment. The RNase H domain of polymerase is associated with KPNA1 for the inhibition of IFN-α signaling [155]. Tembusu virus (TMUV), a member of the Flavivirus genus that primarily causes severe disease in ducks, inhibits type I IFN signaling through its NS5 protein. Functional analyses have identified amino acid residues 37–45 within the N-terminal region of TMUV NS5 as an NLS that mediates interaction with KPNA1, thereby disrupting the host nuclear transport machinery. Specifically, NS5 has been shown to interact with KPNA1, KPNA2, and KPNA5 [220].

While SARS-CoV ORF6 interacts with KPNA2, and N-terminal deletions of KPNA2 do not engage with KPNB1. The C terminus of ORF6 (54–63 aa) is required to inhibit STAT1 nuclear localization. Here, ORF6 recruits KPNA2 and KPNB1 at the ER/Golgi membrane, forming the ORF6:KPNA2:KPNB1 complex, which may compete with the ISGF3:KPNA1:KPNB1 complex for KPNB1. Unavailability of KPNB1 to the ISGF3 complex will block the import of the ISGF3 complex to the nucleuses [221]. Lloviu virus (LLOV) VP24 interacts with KPNA5, competing with phosphorylated STAT1 to abrogate the KPNA5-STAT1 interaction and block IFN signaling [222].

KPNAs are not restricted to IFN signaling pathways; rather, they mediate nuclear transport of diverse NLS-bearing cargo proteins [223]. Consequently, viral targeting of KPNAs has gained more considerable attention, as these proteins may serve as critical gateways for the nuclear import of ISGF3. Moreover, KPNA targeting may represent an effective viral strategy to block the nuclear translocation of non-canonical STAT2-IRF9 complexes and U-ISGF3, thereby impairing the host antiviral state. However, an important question remains: because STAT1 can undergo nuclear translocation through multiple KPNAss, is targeting a single KPNA sufficient to suppress IFN signaling, or can other KPNAs compensate for ISGF3 nuclear import? Such redundancy may underline the strategy employed by the EBOV VP24 protein, which targets multiple KPNAs to inhibit IFN signaling. By blocking several STAT1 nuclear import pathways simultaneously, VP24 minimizes the possibility of compensation by other KPNA family members and achieves more robust suppression of antiviral gene expression [211,218,219]. Although KPNA2 has not been shown to interact with pSTAT1 directly, several viral proteins have been reported to suppress IFN signaling by targeting KPNA2. One such mechanism has been described for the SARS-CoV ORF6 protein, which sequesters KPNA2 and consequently reduces the availability of KPNB1 for association with other KPNAs involved in nuclear transport. Nevertheless, evidence suggests that certain KPNAs can still localize to the nucleus in the absence of KPNB1, raising questions about the precise role of KPNA and KPNB in regulating IFN signaling [224,225]. Therefore, further mechanistic studies are needed to clarify the contribution of KPNA-mediated nuclear transport to IFN responses and to assess the feasibility of targeting these pathways for antiviral therapeutic development. Table 6 provides the mechanisms of virus proteins that are used to downregulate KPNAs.

## 9. Downregulation of CBP

CREB-binding protein (CBP) is a transcriptional co-activator that plays an important role in regulating gene expression through its interaction with the JAK-STAT pathway. It acts as a coactivator for several transcription factors, including STAT proteins. CBP directly interacts with proteins such as STAT1 and STAT3 and enhances their transcriptional activity. In addition, CBP has intrinsic histone acetyltransferase (HAT) activity, which acetylates histones and other proteins. Acetylation relaxes the chromatin structure, making DNA more accessible to the transcriptional machinery, thereby promoting gene transcription [226,227]. This interaction is crucial for the complete transcriptional activation of STAT target genes. The interaction between CBP and STAT proteins amplifies the transcriptional response to JAK-STAT signaling. CBP HAT activity modifies chromatin, enabling a more robust transcriptional response [228]. Thus, CBP plays an important role in the immune response mediated by the JAK-STAT pathway. For example, it enhances the transcriptional activity of STAT1 in response to IFN signaling, which is essential for the antiviral defense. Dysregulation of either JAK-STAT signaling or CBP function can contribute to the development of the disease. In particular, mutations in CBP have been implicated in cancer [229], where altered JAK-STAT signaling is a common feature. Additionally, impaired CBP activity affects immune responses and contributes to inflammatory and autoimmune diseases.

Viral proteins that downregulate CBP and hinder ISG transcription are uncommon (Table 7). For example, SADS-CoV nsp1 induces CBP degradation to inhibit IFN-stimulated gene production and STAT1 acetylation, thereby inhibiting STAT1 dephosphorylation and blocking STAT1 transport out of the nucleus to induce antiviral signaling [80].

## 10. Downregulation of ISRE

The conventional IFN signaling pathway involves the activation of STAT1 and STAT2, as well as the production of the ISGF3 trimer, which binds to genomic sequence patterns known as ISREs and regulates the transcription of ISGs.

A few viral proteins (Table 8) have been reported to downregulate the IFN signaling pathway interacting with ISRE. For example, the Cap of PCV3 interacts with the ISRE promoter and prevents ISRE from interacting with the ISGF3 mimic, IRF9-S2C [185]. The processivity factor for DNA polymerase UL42 of PRV and HSV-1 directly interacts with ISRE and prevents ISGF3 from binding to ISRE for effective gene transcription, and this relationship requires four conserved amino acids (K124, R196, Q279, and R280) of the UL42 DNA-binding site in UL42 of PRV and R113, R182, R279, and R280 of the UL42 DNA-binding site in the UL42 of HSV-1 [230].

## 11. Conclusion and Future Expectations

Over the past 25 years, researchers have mapped out how viral IFN antagonists shut down host immune response. However, there is a significant catch: most of what we know comes from in vitro transfection experiments, not from studying viruses in an infectious setting. This means that we still do not fully understand the role of these proteins in viral infection. To clarify this, we need to study viruses in more realistic settings using live animal models, recombinant viruses, and genetically modified animals. Relying more heavily on mutant viruses during actual infection is essential for determining the function of each viral protein. In addition, several experimental factors muddy our understanding. For instance, lab-passaged virus strains often drift away from clinical isolates because RNA viruses mutate quickly due to their error-prone polymerases. Many commonly used cell lines also have hidden problems with innate immune signaling. There are also methodological issues: knockdowns do not behave the same as full knockouts, and transient overexpression can create artificial protein interactions that do not occur naturally. To make matters worse, very few studies confirm that viral and host proteins bind directly using biochemical assays, leaving it unclear whether the observed effects result from direct binding between the two proteins or involve other host factors acting as intermediaries.

The development of recombinant live-attenuated vaccines with targeted gene deletions represents one of the most effective strategies for controlling highly virulent diseases. By creating recombinant viruses with specific IFN antagonist genes deleted (such as ASFV-ΔI7L, -ΔMGF360-10L) [79,151], we can accomplish three things at once: test whether those proteins are actually virulence factors, determine whether those proteins are essential for virus replication, and generate safer, attenuated vaccine candidates that still trigger strong immune responses. This approach bridges the gap between laboratory-based mechanistic studies and the actual events occurring in a living host. It also helps identify which viral proteins are critical for pathogenicity. Importantly, building these vaccines from clinical isolates rather than lab-adapted strains ensures that they reflect the genetics and immune evasion tactics of viruses circulating in nature, which should make them more effective at protecting against real-world infections.

To restrict immune evasion by viral proteins, another strategy is to use small molecules that activate immune adapters and target viral virulence factors. As excessive IFN signaling can trigger detrimental inflammatory responses and associated adverse effects, directly targeting JAK-STAT pathway adaptors to enhance their expression may not be an optimal therapeutic strategy for treating viral infections. This is because the JAK-STAT pathway functions as a double-edged sword, mediating both antiviral immunity and immunopathology [231,232]. Thus, virus protein-targeted strategies are more attractive because they can restore antiviral signaling without directly manipulating the host immune system. Blocking viral–host protein interactions, targeting viral enzymatic activities, destabilizing viral antagonists, and structure-guided drug design are the best strategies for designing small-molecule drugs and peptides. However, several issues remain, such as interaction surfaces that can be large and difficult to target with small molecules, and rapid viral evolution against drugs may lead to resistance, such as RSV [233].

As discussed in previous chapters, several studies have identified key amino acid motifs and residues that govern the interaction between viral proteins and host IFN regulators and contribute to their enzymatic functions. For example, Hagmaier et al. reported that glycine 125 of the V protein of NiV is critical for IFN antagonistic activity, and mutation of this residue disrupts interactions with both STAT1 and STAT2 [234]. Targeting these interaction sites with small-molecule drugs could prove effective in blocking viral immune evasion strategies [235]. However, one of the major limitations of the current literature is that many viral proteins involved in IFN antagonism remain insufficiently characterized in terms of PTMs. This gap constrains our understanding of how these proteins function and are regulated during infection. Many viral antagonists do not rely on enzymatic activity alone; instead, they suppress IFN signaling by recruiting E3 ligases for protein degradation or activating caspase-mediated cleavage. These observations suggest that therapeutic strategies should extend beyond conventional enzymatic active site inhibition or simple blockade of protein–protein interactions. A more effective approach may be to target the maturation process of viral proteins, as their activity depends on PTMs, such as glycosylation, phosphorylation, palmitoylation, N-myristoylation, and disulfide bond formation. Disruption of these essential modification events can compromise viral protein stability, localization, and function. Accordingly, future studies should prioritize the structural and functional characterization of these viral proteins, with particular attention to residues and modification sites that are critical for maturation and activity and can be targeted by small molecules to block immune evasion. For example, phosphorylation of Y98 is critical for the immune evasion function of ASFV I7L, as mutation of this residue attenuates its ability to suppress the innate immune response. [151]. The following question to be addressed is the structures of virulence factors that remain undefined for molecular docking and pharmacophore modelling to discover small molecule hits. Defining structures through crystallography is often challenging; instead, cryo-EM or AlphaFold predictions can be utilized [219,236,237]. Nevertheless, the functional redundancy of viral proteins, particularly in large viruses such as ASFV, may limit the effectiveness of small molecules targeting a single viral protein, as other viral factors can compensate for their roles in immune evasion [71,72,73]. Thus, a deeper understanding of these features will not only clarify how viruses evade IFN signaling but also reveal new vulnerabilities that may be exploited for antiviral drug development.

## Figures and Tables

**Table 1:** IFNR downregulation by virus proteins.

Protein	Virus	Virulence Factor	Function	Ref.
IFNAR1	EBV	LMP2A	Degradation	[60]
		LMP2B		
	IAV	HA	Polyubiquitination and proteasomal/ lysosomal degradation	[61]
	IAV	HA	Casein Kinase 1-mediated degradation	[62]
	PRV	UL50	Lysosomal degradation	[64]
	MHV-68	ORF54	Degradation	[65]
	ASFV	p22	Autophagic degradation	[38]
	HBV	X	Expression inhibition	[68]
	LGTV	NS5	Physical interaction with prolidase to prevent surface expression	[69]
	EV71	2A^pro^	Expression inhibition	[70]
	EV-A71	2A^pro^	Translation inhibition	[46]
	ASFV	B318L	IFNAR1-TYK2 interaction inhibition	[71]
	ASFV	H240R	IFNAR1-TYK2 interaction inhibition	[72]
	ASFV	pK205R	IFNAR1-TYK2 interaction inhibition	[73]
	LSDV	LSDV122	IFNAR1 and IFNAR2 interaction inhibition	[75]
IFNAR2	ASFV	B125R	Autophagic degradation	[66]
	ASFV	B318L	IFNAR2 and JAK1 interaction inhibition	[71]
	ASFV	H240R	IFNAR2 and JAK1 interaction inhibition	[72]
	ASFV	pK205R	IFNAR2 and JAK1 interaction inhibition	[73]
	HSV-1	UL36USP	IFNAR2 and JAK1 interaction inhibition	[74]
	LSDV	LSDV122	IFNAR1 and IFNAR2 interaction inhibition	[75]
IFNGR1	EBV	LMP2A	Degradation	[60]
		LMP2B		
	IAV	HA	Casein Kinase 1 mediated ubiquitination & lysosomal degradation	[62]
	KHSV	K3 and K5	Ubiquitination and degradation	[52]
	EBV	BZLF1	Transcription inhibition	[43]

**Table 2:** Downregulation of JAKs by virus proteins.

Protein	Virus	Virulence Factor	Function	Ref.
JAK1	IAV	PB2	K48 ubiquitination and proteasomal degradation	[53]
	HPAI	PB2		
	ZIKV	NS2B3	Proteasomal degradation	[76]
	ASFV	MGF505-7R	Ubiquitination and proteasomal degradation	[77]
	FMDV	VP3	Lysosomal degradation	[78]
	ASFV	MGF360-10L	K48 ubiquitination and proteasomal degradation	[79]
	SADS-CoV	nsp1	K11 and K48 ubiquitination and proteasomal degradation	[80]
	IBV	Nsp14	Autophagic degradation	[81]
	HMPV	SH	Ubiquitination and Proteasomal degradation	[82]
	SVA	3D	K48 ubiquitination and proteasomal degradation	[83]
	MV	V	Physical interaction	[84]
	ISKNV	ORF103R	Physical interaction	[85]
JAK2	ASFV	MGF505-7R	Proteasomal/lysosomal/autophagy degradation	[77]
	FMDV	VP3	Physical interaction	[78]
TYK2	HPV-18	E6	Physical interaction	[86]
	TBEV	NS5	Physical interaction	[87]
	LIV			
	EBV	LMP-1	Phosphorylation inhibition	[54]

**Table 3:** STAT1 downregulation by virus proteins.

Virus Family	Virus	Virulence Factor	Function	Ref.
Paramyxoviridae	SV5 virus	V	Proteasomal degradation	[106,107]
	SV5 virus	V	Proteasomal degradation	[108]
	SV5 virus	V	Polyubiquitination and proteasomal degradation	[109,110]
	NiV	V	Alters the STAT protein subcellular distribution	[111]
	NiV	V	Co-localization	[112]
	NiV	V	Co-localization	[113]
	NiV	P		
	NiV	W		
	NiV	P	Sequestration to viral inclusion bodies	[114]
	SeV	C	Physical interaction	[55]
	SeV	C	Phosphorylation and dephosphorylation inhibition	[115]
	SeV	C′, C, Y1, Y2	Physical interaction	[116]
	SeV	C	Physical interaction	[117]
	HeV	V	Physical interaction	[118]
	Neurovirulent CDV A75/17 strain	V	Physical interaction	[119]
	MuV	V	Polyubiquitination and proteasomal degradation	[120]
	MuV	V	Degradation and inhibition of phosphorylation	[121]
	MV	P	Phosphorylation inhibition	[122]
	MV	V	Physical interaction	[84]
	HPIV1	C	Physical interaction	[123]
	NDV	V	Proteasomal degradation	[124]
Coronaviridae	SARS-CoV-2	N	Physical interaction	[125]
	SARS-CoV-2	NSP13	Physical interaction	[126]
	SARS-CoV-2	NSP13	Physical interaction	[127]
	SARS-CoV-2	S	Physical interaction	[128]
	SARS-CoV-2	S	Physical interaction	[129]
	SARS-CoV-2	nsp5	Autophagic degradation	[130]
	SARS-CoV-2	ORF6	Physical interaction	[131]
	PEDV	nsp7	Physical interaction	[132]
	PDCoV	NS7a	Phosphorylation inhibition	[133]
	PDCoV	N	Physical interaction	[134]
Picornaviridae	FMDV	Lbpro	Cleavage	[135]
	EV71	3D	mRNA expression & phosphorylation inhibition	[136]
	EV71	2A	Phosphorylation inhibition	
	EV-D68	3C protease	Cleavage	[137]
	SVV	3C^pro^	Degradation	[40]
	SVV	3C^pro^	Cleavage and degradation	[41]
Herpesviridae	HSV-2	ICP22	Ubiquitination and proteasomal degradation	[138]
	BoHV-1	VP8	Physical interaction	[139]
	BoHV-1	UL41	mRNA cleavage	[45]
Flaviviridae	HCV	Full-length HCV, HCV core/E1/E2 & NS3-4A	Proteasomal degradation	[140]
	HCV	Core	Physical interaction	[141]
	HCV	NS5A	Phosphorylation inhibition	[142]
	HCV genotype 1	NS5A	Physical interaction	[143]
	ZIKV-INMI1	NS4B	Phosphorylation inhibition	[144]
	ZIKV	NS2A	Proteasomal degradation	[145]
Rhabdoviridae	RABV	P	Nuclear translocation inhibition	[56]
	RABV	P	Physical interaction	[146]
	RABV	P	Nuclear translocation inhibition DNA binding inhibition	[147]
Asfaviridae	ASFV	MGF360-9L	Degradation via apoptosis pathway	[148]
	ASFV	A104R	Phosphorylation inhibition	[149]
	ASFV	F778R	Nuclear translocation inhibition	[150]
	ASFV	I7L	Physical interaction	[151]
	ASFV	MGF 505-4R	Phosphorylation inhibition	[152]
Poxviridae	VV virus	VH1	Dephosphorylation	[153]
Hepadnaviridae	HBV	Polymerase	Nuclear translocation inhibition	[154]
	HBV	Polymerase	Phosphorylation inhibition	[155]
Filoviridae	EBOV	VP24	Physical interaction	[156]
	EBOV	VP35	Phosphorylation inhibition	[157]
Phenuiviridae	SFTSV	NSs	Physical interaction	[158]
Reoviridae	RV	NSP1	Phosphorylation inhibition	[159]
Togaviridae	CHIKV	nsP2	Promotion of nuclear export	[160]
Pneumoviridae	RSV	NS1	Physical interaction	[161]
Papillomaviridae	HPV	E1	Physical interaction	[57]

**Table 4:** STAT2 downregulation by virus proteins.

Virus Family	Virus	Virulence Factor	Function	Ref.
Paramyxoviridae	NiV	V	Alters the STAT protein subcellular distribution	[111]
	NiV	V	Physical interaction	[112]
	hPIV2	V	Proteasomal degradation	[107]
	hPIV2	V	Polyubiquitination and proteasomal degradation	[109]
	hPIV2	V	Degradation	[165]
	HeV	V	Physical interaction	[118]
	MuV	V	Phosphorylation inhibition	[121]
	MV	V	Physical interaction	[166]
	Neurovirulent CDV A75/17 strain	V	Physical interaction	[119]
Coronaviridae	SARS-CoV-2	N	Physical interaction	[125]
	SARS-CoV-2	ORF7a	Phosphorylation inhibition	[167]
	SARS-CoV-2	NSP1	Translation inhibition	[47]
	SARS-CoV-2	S	Physical interaction	[129]
	PDCoV	NS7a	Physical interaction	[133]
	SADS-CoV	E	Autophagy degradation	[39]
Flaviviridae	DENV	NS5	Ubiquitination & proteasomal degradation	[168]
	DENV	NS5	Degradation	[169]
	YFV	NS5	Physical interaction	[170]
	ZIKV	NS5	Physical interaction	[171]
	ZIKV	NS5	Ubiquitination and proteasomal degradation	[172]
	ZIKV	NS2A	Proteasomal degradation	[145]
	WNV-KUN	NS2A, NS2B3, NS4A, NS4B	Nuclear translocation inhibition	[173]
Picornaviridae	FMDV	Lbpro	Cleavage	[135]
	FMDV	3D polymerase	Physical interaction	[174]
	SVV	3C^pro^	Cleavage and degradation	[40]
	SVV	3C^pro^	Cleavage and degradation	[41]
Phenuiviridae	SFTSV	NSs	Physical interaction	[175]
	HRTV	NSs	Physical interaction	[176]
Herepesviridae	HSV-2	ICP22	Ubiquitination and proteasomal degradation	[138]
	EBV	BGLF2	K48 ubiquitination & proteasomal degradation	[177]
	HCMV	IE1	Physical interaction	[178]
Asfaviridae	ASFV	pS273R	K48 ubiquitination and degradation	[179]
	ASFV	pI215L	Ubiquitination and degradation	[180]
	ASFV	MGF360-9L	Proteasomal Degradation	[148]
	ASFV	B475L	Physical interaction	[181]
	ASFV	MGF 505-4R	Phosphorylation inhibition	[152]
Poxviridae	MPXV	PoxS	Physical interaction	[182]
	VV	C6	Physical interaction	[183]
Arteriviridae	PRRSV	nsp11	K48 ubiquitination and proteasomal degradation	[184]
Circoviridae	PCV3	Cap	Physical interaction	[185]
Reoviridae	BTV	NS3	Autophagic/lysosomal degradation	[186]
Papillomaviridae	HPV16	E4	Physical interaction	[187]
Rhabdoviridae	RABV	P	Physical interaction	[146]
Pneumoviridae	RSV	NS1 & NS2	Ubiquitination & proteasomal degradation	[188]

**Table 5:** IRF9 downregulation by virus proteins.

Protein	Virus	Virulence Factor	Function	Ref.
IRF9	Simian Varicella Virus	ORF63	Proteasomal degradation	[202]
	VZV		Degradation	
	ASFV	pI215L	Autophagy/lysosomal degradation	[203]
	ASFV	MGF360-12L	Degradation	[204]
	HSV-2	ICP22	Ubiquitination and proteasomal degradation	[138]
	RV	NSP1	Degradation	[205]
	EV71	3C^pro^	Cleavage	[206]
	SVV	3C^pro^	Degradation	[40]
	PBoV	NP1	Physical interaction	[207]
	PRRSV	nsp11	Physical interaction	[208]
	PDCoV	NS7a	Physical interaction	[133]
	SARS-CoV-2	S	Physical interaction	[129]
	HPV16	E4	Physical interaction	[187]
	T1L	μ2	Nuclear accumulation	[209]
	PRV	EP0	Transcription inhibition	[44]

**Table 6:** KPNA downregulation by virus proteins.

Protein	Virus	Virulence Factor	Function	Ref.
KPNA1	PRRSV	Nsp1β	Ubiquitination & proteasomal degradation	[213]
	FMDV	3C^pro^	Degradation	[214,215]
	EV-A71	2B	Degradation	[216]
	HPV	E6	Ubiquitination and proteasomal degradation	[217]
	SVV	3C^pro^	Degradation	[40]
	EBOV	VP24	Physical interaction	[211,218]
	SARS-CoV-2	ORF6	Physical interaction	[131]
	HBV	Polymerase	Physical interaction	[155]
	TMUV	NS5	Physical interaction	[220]
KPNA2	PDCoV	N	Lysosomal degradation	[134]
	SARS-CoV	ORF6	Physical interaction	[221]
	SARS-CoV-2	ORF6	Physical interaction	[131]
	TMUV	NS5	Physical interaction	[220]
KPNA5	EBOV	VP24	Physical interaction	[211,219]
	LLOV	VP24	Physical interaction	[222]
	TMUV	NS5	Physical interaction	[220]
KPNA6	EBOV	VP24	Physical interaction	[211]

**Table 7:** CBP downregulation by virus proteins.

Protein	Virus	Virulence Factor	Function	Ref.
CBP	SADS-CoV	nsp1	Degradation	[80]

**Table 8:** ISRE downregulation by virus proteins.

Protein	Virus	Virulence Factor	Function	Ref.
ISRE	PCV3	Cap	Physical interaction	[185]
	PRV	UL42	Physical interaction	[230]
	HSV-1	UL42		

## Data Availability

No new data were created or analyzed in this study. Data sharing is not applicable to this article.

## Abbreviations

The following abbreviations are used in this manuscript:

IFN	Interferon
PRRs	Pattern recognition receptors
ISGs	IFN-stimulated genes
JAK-STAT	Janus Kinase-signal transducer and activator of transcription
IL-10	Interleukin-10
HCV	Hepatitis C virus
WNV	West Nile virus
YFV	Yellow fever virus
VSV	Vesicular stomatitis virus
DENV	Dengue virus
HSV	Herpes simplex virus
ZIKV	Zika virus
JEV	Japanese encephalitis virus
PTMs	Post-translational modifications
IFNRs	IFN receptors
IFNAR	IFN receptor
IFNAR	IFN alpha receptor
IFNGR	IFN gamma receptor
IFNLR	IFN lambda receptor
IL-10R2	the interleukin-10 receptor β subunit
TYK2	Tyrosine kinase 2
JAK1	Janus kinase 1
IRF9	Interferon regulatory factor 9
ISGF3	ISG factor 3
GAF	Gamma interferon activation factor
GAS	Gamma-activated sites
EBV	Epstein–Barr Virus
PRV	Pseudorabies Virus
BoHV-1	Bovine alpha herpesvirus 1
EV-A71	Enterovirus A71
eIF4GI	Eukaryotic initiation factor 4GI
SARS-CoV-2	Severe acute respiratory syndrome coronavirus 2
LMP2A	Latent membrane protein 2A
LMP2B	Latent membrane protein 2B
IAV	Influenza A virus
HA1	HA subunit 1
CK1α	Casein kinase 1 α
HBV	Hepatitis B virus
HBX	HBV X protein
LGTV	Langat virus
PEDP	Prolidase
MHV-68	Gammaherpesvirus-68
EV71	Enterovirus 71
ASFV	African swine fever virus
GGPPS	Transgeranylgeranyl-diphosphate synthase
TAX1BP1	Tax1-binding protein 1
TMD	Transmembrane domain
LSDV	Lumpy skin disease virus
KSHV	Kaposi’s sarcoma-associated herpesvirus
JH	JAK homology
MV	Measles virus
HPAI	Highly pathogenic avian influenza
vSOCS	viral suppressor of cytokine signaling SOCS
ISKNV	Infectious spleen and kidney necrosis virus
FMDV	Foot and mouth disease virus
HERC5	HECT and RLD domain-containing E3 ubiquitin protein ligase 5
SADS-CoV	Swine acute diarrhoea syndrome coronavirus
IBV	Avian infectious bronchitis virus
SH	Small hydrophobic
HMPV	Human metapneumovirus
SVA	Senecavirus A
HPV	Human papillomavirus
LMP-1	Latent membrane protein 1
TBEV	Tick-borne encephalitis virus
LIV	Louping ill virus
RdRp	RNA polymerase
TAD	Transcription-activation domain
DBD	DNA-binding domain
NTD	N-terminal domain
PIAS	Protein inhibitor of activated STAT
EBOV	Ebola virus
SV5	Simian virus 5
PIV5	Parainfluenza virus 5
DDB	UV damage-specific DNA binding protein
NiV	Nipah virus
NES	Nuclear export signal
SeV	Sendai virus
VV	Vaccinia virus
HeV	Hendra virus
CDV	Canine distemper virus
MuV	Mumps virus
RABV	Rabies virus
HPIV1	Human parainfluenza virus type 1
SFTSV	Severe fever with thrombocytopenia syndrome virus
NDV	Newcastle disease virus
CHIKV	Chikungunya Virus
RBD	receptor binding domain
PEDV	Porcine epidemic diarrhoea virus
KPNA1	Karyopherin α 1
RV	Rotavirus
PKC-δ	Protein kinase C-δ
SVV	Seneca Valley Virus
EV-D68	Respiratory enterovirus D68
PDCoV	Porcine delta coronavirus
RSV	Respiratory Syncytial Virus
HCMV	Human cytomegalovirus
IE1	Immediate-early 1 protein
UBR4	E3 component n-recognin 4
IBs	Inclusion bodies
HRTV	Heartland virus
Cap	Capsid protein
PCV3	Porcine circovirus 3
BTV	Bluetongue virus
3Cpro	3C protease
MPXV	Monkeypox virus
PoxS	Poxin-schlafen
OPTN	Optineurin
VZV	Varicella-zoster virus
NendoU	Nidovirus-specific endoribonuclease
EP0	Early protein 0
PBoV	Porcine bocavirus
KPNB	Karyopherin β
IBB	Importin-β binding
TMUV	Tembusu virus
LLOV	Lloviu virus
CBP	CREB-binding protein
HAT	Histone acetyltransferase
ISREs	IFN-stimulated response elements
